# Prognostic impact of alternative splicing-derived hMENA isoforms in resected, node-negative, non-small-cell lung cancer

**DOI:** 10.18632/oncotarget.2609

**Published:** 2014-10-21

**Authors:** Emilio Bria, Francesca Di Modugno, Isabella Sperduti, Pierluigi Iapicca, Paolo Visca, Gabriele Alessandrini, Barbara Antoniani, Sara Pilotto, Vienna Ludovini, Jacopo Vannucci, Guido Bellezza, Angelo Sidoni, Giampaolo Tortora, Derek C. Radisky, Lucio Crinò, Francesco Cognetti, Francesco Facciolo, Marcella Mottolese, Michele Milella, Paola Nisticò

**Affiliations:** ^1^ Department of Medical Oncology, Regina Elena National Cancer Institute, Rome, Italy; ^2^ Medical Oncology, Azienda Ospedaliera Universitaria Integrata, University of Verona, Verona, Italy; ^3^ Laboratory of Immunology, Regina Elena National Cancer Institute, Rome, Italy; ^4^ Biostatistics and Scientific Direction, Regina Elena National Cancer Institute, Rome, Italy; ^5^ Department of Pathology, Regina Elena National Cancer Institute, Rome, Italy; ^6^ Thoracic Surgery, Regina Elena National Cancer Institute, Rome, Italy; ^7^ Medical Oncology, University of Perugia, Perugia, Italy; ^8^ Department of Thoracic Surgery, University of Perugia, Perugia, Italy; ^9^ Institute of Pathological Anatomy and Histology, University of Perugia, Perugia, Italy; ^10^ Mayo Clinic Cancer Center, Jacksonville, FL, USA

**Keywords:** Lung cancer, Splicing, Biomarkers

## Abstract

Risk assessment and treatment choice remain a challenge in early non-small-cell lung cancer (NSCLC). Alternative splicing is an emerging source for diagnostic, prognostic and therapeutic tools. Here, we investigated the prognostic value of the actin cytoskeleton regulator hMENA and its isoforms, hMENA^11a^ and hMENAΔv6, in early NSCLC.

The epithelial hMENA^11a^ isoform was expressed in NSCLC lines expressing E-CADHERIN and was alternatively expressed with hMENAΔv6. Enforced expression of hMENAΔv6 or hMENA^11a^ increased or decreased the invasive ability of A549 cells, respectively. hMENA isoform expression was evaluated in 248 node-negative NSCLC. High pan-hMENA and low hMENA^11a^ were the only independent predictors of shorter disease-free and cancer-specific survival, and low hMENA^11a^ was an independent predictor of shorter overall survival, at multivariate analysis. Patients with low pan-hMENA/high hMENA^11a^ expression fared significantly better (*P*≤0.0015) than any other subgroup. Such hybrid variable was incorporated with T-size and number of resected lymph nodes into a 3-class-risk stratification model, which strikingly discriminated between different risks of relapse, cancer-related death, and death. The model was externally validated in an independent dataset of 133 patients.

Relative expression of hMENA splice isoforms is a powerful prognostic factor in early NSCLC, complementing clinical parameters to accurately predict individual patient risk.

## INTRODUCTION

Lung cancer is the leading cause of cancer death worldwide regardless of gender [1], with non-small cell lung cancer (NSCLC) accounting for 80% of cases, and overall survival remains poor, particularly for the majority of lung cancer patients who are identified at later stages of disease [2]. While improved methods for screening high-risk individuals holds the possibility for identification of disease at earlier stages, the improved identification of early stage disease has not yet led to substantially improved outcomes for these patients as a group [3]. One reason for this is the lack of reliable prognostic indicators for early stage NSCLC, particularly for patients with node-negative (N0) disease. Indeed, even radically resected stage I NSCLC carries a high risk of recurrence, which is not substantially modified by adjuvant treatment [4]. Research efforts have focused on a large number of potential clinical and biological prognostic and predictive factors [5-9]. However, genomic and proteomic signatures have not yet entered routine clinical practice due to costs, lack of reproducibility, and inability to clearly identify potential therapeutic targets [10, 11].

Alternative splicing has emerged as an additional layer of gene regulation and differential protein isoform expression represents a potential biomarker of diagnosis, prognosis, invasiveness, and response to therapy in different tumors, including lung cancer [12]. Human MENA (hMENA), a member of the Ena/Vasp family of actin regulatory proteins [13], has been identified as a regulator of cell invasiveness and metastatic potential according to the expression of alternative splice isoforms. hMENA can be expressed along with variable levels of two splice variant-derived isoforms, hMENA^11a^ and hMENAΔv6, which have been shown to have opposite regulatory functions, with hMENA^11a^ acting to suppress cancer cell invasion and hMENAΔv6 stimulating the invasive phenotype [14]. Studies using experimental models have provided insight into how hMENA isoform expression is controlled by the epithelial splicing regulatory proteins (ESRP1/2) [15] and, in turn, how relative expression levels of hMENA isoforms could directly drive changes in the cellular phenotype [14, 16, 17]. Here, we investigate hMENA isoform expression in lung cancer cells as a potential prognostic biomarker of progression to metastatic disease. Our results indicate that, in the setting of early stage, node negative NSCLC, hMENA alternative splicing represents a powerful prognostic indicator.

## RESULTS

### Alternative expression of hMENA isoforms regulates NSCLC invasive capacity

**Figure 1:**
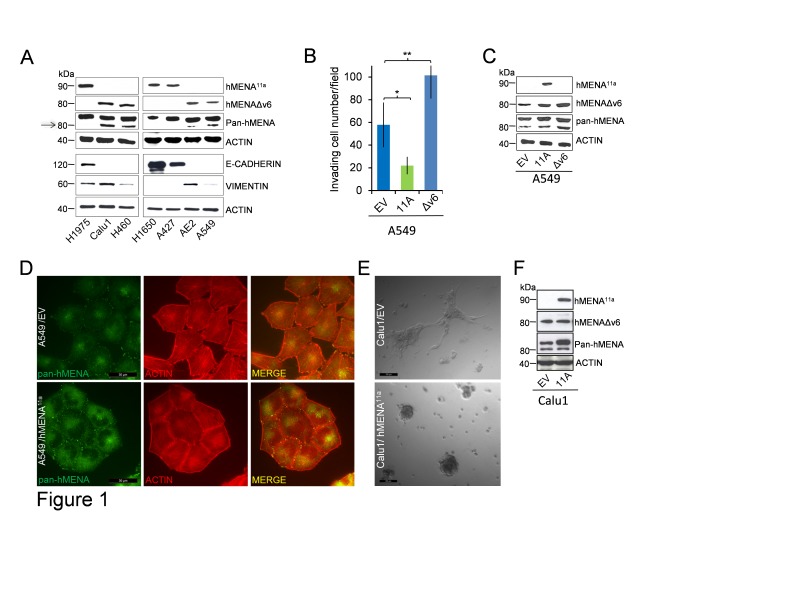
hMENA^11a^ defines an epithelial phenotype and is expressed alternatively to hMENAΔv6 isoform in lung cancer cell lines The isoforms have opposite and antagonistic roles in lung cancer cell invasion and affect cell morphology in 2D and 3D cultured cells. (A) WB analysis of lysates of lung tumor cell lines with hMENA isoform specific and pan-hMENA [which recognizes all hMENA isoforms, with apparent molecular weights of 90kDa, hMENA^11a^, 88kDa, hMENA and 80kDa, hMENAΔv6 (arrow)] and E-CADHERIN and VIMENTIN antibodies, indicating a strong correlation between hMENA^11a^and E-CADHERIN expression. (B) Matrigel invasion assays of A549 cells transfected with the empty vector (EV), with hMENA^11a^ (11A), or with hMENAΔv6 (Δv6). The invasive ability was measured using Matrigel coated transwell filters towards a serum gradient. The assay was repeated three times and performed in triplicate each time. * Significantly different as determined by Student t tests p=0.027; **p=0.004. (C) WB analysis of A549 cells transfected with the empty vector, with hMENA^11a^, or with hMENAΔv6, using hMENA isoform-specific Abs or pan-hMENA Ab. (D) Immunofluorescence analysis of A549 cells transfected with the empty vector or with hMENA^11a^ using a pan-hMENA mAb, indicating a colocalization of hMENA isoforms (green) with phalloidin stained actin filaments (red). Cells were imaged using immunofluorescence microscopy DMIRE2 (Leica Microsystems) and processed using FW4000 Software. Magnification: 63X. Scale Bar: 30 μm. (E) Representative phase-contrast images of Calu1 cells transfected with the empty vector (EV) or hMENA^11a^ (11A) and grown for 72h in 3D lrECM. Magnification: 20X. Scale Bar: 100μm. (F) WB analysis of Calu1 cells transfected with the empty vector or with hMENA^11a^, using hMENA isoform-specific Abs or pan-hMENA Ab.

We evaluated hMENA isoform expression in a panel of human lung cancer cell lines (characteristics of these cell lines are reported in Table S1), using Western blot analysis with two isoform-specific Abs recently characterized by our group [14, 16]. hMENA^11a^ and hMENAΔv6 isoforms were alternatively expressed, with hMENA^11a^expressed in E-CADHERIN-positive cell lines, and hMENAΔv6 expressed in E-CADHERIN-negative, VIMENTIN positive cell lines (Fig. 1A). Using the pan-hMENA Ab, which recognizes all hMENA isoforms, we showed that the hMENA 88kDa is expressed along with hMENA^11a^ or hMENAΔv6 in all the cell lines examined (Fig. 1A).

To define how hMENA isoform expression could drive phenotypic characteristics in NSCLC cells, we overexpressed hMENA^11a^ or hMENAΔv6 in hMENAΔv6-positive A549 cells. We found increased invasion into Matrigel in hMENAΔv6-transfected A549 cells and decreased invasion in hMENA^11a^–transfected cells (Fig. 1B-C). Such effect on invasive capacity is not accompanied by reduced proliferative ability, since hMENA^11a^ overexpression in A549 cells resulted in a significantly higher [^3^H] Thymidine incorporation (Fig. S1A), as we have already reported in breast cancer [16]. Although not associated with changes in the expression of EMT markers, such as E-CADHERIN or VIMENTIN (Fig. S1B), the altered cellular invasion observed in transiently transfected A549 cells was accompanied by a modification of the actin cytoarchitecture and cell morphology, with hMENA^11a^transfected cells growing in more tightly packed colonies (Fig. 1D). This effect is even more evident in the ‘mesenchymal’ Calu1 cells transfected with hMENA^11a^and grown in 3D laminin-rich Extracellular Matrix (3D lrECM) (Fig. 1E-F) [18]. By contrast, overexpression of hMENAΔv6 in hMENA^11a^-positive H1975 cells only slightly affected cell invasiveness (Fig. S2).

### hMENA isoform expression predicts recurrence and survival in early NSCLC

**Figure 2:**
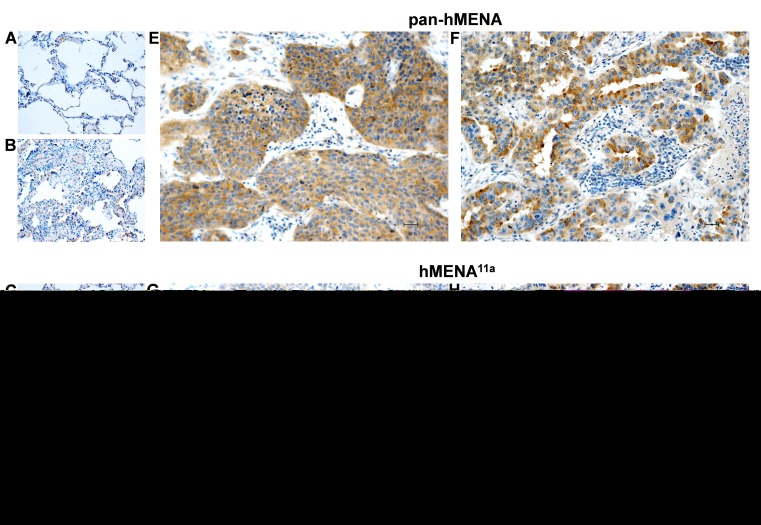
Pan-hMENA and hMENA protein expression in normal lung tissue, atypical adenomatous lung hyperplasia and lung carcinomas by immunohistochemistry Non-neoplastic alveolar structures are negative for both pan-hMENA (A) and isoform-specific hMENA^11a^mAb (C) staining, whereas atypical adenomatous hyperplasia (B) shows a weak cytoplasmic pan-hMENA staining (score1+) and no immunoreactivity for hMENA^11a^(D). Panels E-H show two representative lung cancer cases: a poorly differentiated squamous lung carcinoma displaying a strong cytoplasmic pan-hMENA positivity (score 3+) and no hMENA^11a^immunoreactivity (E, G) and a moderately differentiated adenocarcinoma displaying a strong positivity (score 3+) both for pan-hMENA and hMENA^11a^(F, H). Scale bar 30 μm.

**Figure 3:**
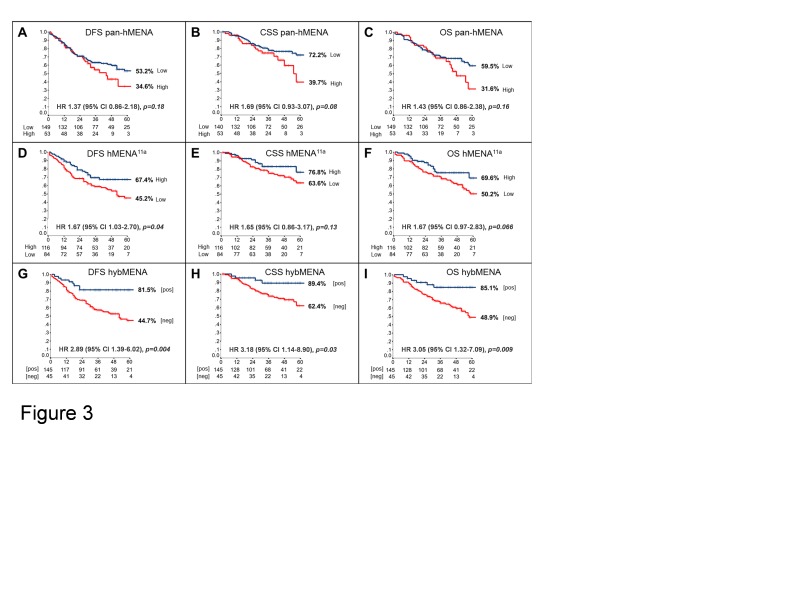
Prognostic impact of hMENA isoform expression in node-negative NSCLC Kaplan-Meier analysis of (A) Disease-Free- (DFS), (B) Cancer-Specific- (CSS), and (C) Overall-Survival (OS) of resected, node-negative, NSCLC patients according to dichotomized pan-hMENA (A-C), hMENA^11a^ (D-F), and hybMENA (G-I) expression. HR: Hazard Ratio; CI: confidence intervals; p-value: log-rank test.

To assess the prognostic potential of hMENA splice isoforms in early NSCLC, we analyzed a series of 248 node-negative patients, resected with curative intent. Relevant clinico-pathological patient characteristics are reported in Table S2. At a median follow-up of 36 months (range: 1-96), 86 deaths (59 due to cancer, 27 due to other causes) and 78 recurrences had occurred. IHC revealed that normal lung tissue, including bronchial epithelial and alveolar cells, did not stain with either pan-hMENA- or hMENA^11a^-specific Abs. Conversely, approximately half of the lung carcinomas stained positive for pan-hMENA and/or hMENA^11a^ (Fig. 2 and Fig.S3). Expression of the pro-invasive hMENAΔv6 isoform could not be directly assessed, due to the lack of IHC-validated antibodies. Among the bio-molecular factors tested (EGFR gene mutations; EGFR, pAKT, HER-2, ERα, ERβ1, ERβ2, E-CADHERIN, VIMENTIN, pan-hMENA, and hMENA^11a^ protein expression), pan-hMENA and hMENA^11a^ expression, evaluated as continuous variables derived from the product of staining intensity and percentage of positive cells, were the only significant predictors of disease-free survival (DFS) and cancer-specific survival (CSS) (p≤0.08) and DFS and overall survival (OS) (p≤0.07), respectively, at multivariate analysis. Expression of hMENA and hMENA^11a^was then dichotomized based on optimal cut-offs identified by maximally selected log-rank statistics (Fig. S4A-B). No significant correlation between pan-hMENA or hMENA^11a^ expression and clinico-pathological characteristics was observed (Table S3). Multivariate analysis using categorical variables identified T-size (T2-4), the number of resected lymph nodes (<10), high pan-hMENA and low hMENA^11a^expression as independent predictors of shorter DFS; T-size, high pan-hMENA and low hMENA^11a^expression were independent predictors of shorter CSS; T-size, the number of resected lymph nodes and low hMENA^11a^expression were independent predictors of shorter OS (Table 1). By Kaplan-Meier analysis, patients with high pan-hMENA expression had a non-significant trend towards a worse outcome (Fig. 3A-C), while patients with high hMENA^11a^ expression had a significant and borderline significant advantage in DFS (p=0.03) and OS (p=0.056), respectively, and a non-significant trend towards better CSS (Fig. 3D-F). Table S4 summarizes 3- and 5-year outcomes according to selected clinical/molecular predictors. The impact of pan-hMENA and hMENA^11a^ expression on DFS was internally validated by bootstrap re-sampling analysis with 78% and 83% replication rates for pan-hMENA and hMENA^11a^, respectively.

**Table 1:** Multivariate analysis according to outcome

	DFS (HR 95% CI), p-value	CSS (HR 95% CI), p-value	OS (HR 95% CI), p-value
T-size*	1.76 (1.00-3.09), p=0.05	2.56 (1.24-5.28), p=0.01	1.98 (1.10-3.58), p=0.02
RN	1.84 (1.16-2.94), p=0.01	n.s.	1.83 (1.10-3.05), p=0.02
pan-hMENA	1.67 (1.00-2.81), p=0.05	2.34 (1.22-4.51), p=0.01	n.s.
hMENA11a	1.85 (1.10-3.12), p=0.02	1.88 (0.93-3.82), p=0.08	1.68 (0.97-2.91), p=0.06

DFS: disease free survival; HR: hazard ratio; CI: confidence intervals; CSS: cancer specific survival; OS: overall survival; n.s.: not significant; T-size: tumor size; RN: resected lymph nodes.

* According to the TNM classification of malignant tumors, 7th edition.

### Creation of a dichotomized, hybrid hMENA/hMENA^11a^ variable

Within the four subgroups of patients identified by the possible combinations of high and low expression of pan-hMENA and hMENA^11a^ (a representative case is reported in Fig. S4C-D), patients with low pan-hMENA/high-hMENA^11a^ expression (hereafter referred to as hybMENA, for hybrid MENA positive) fared significantly better (p<0.0015 for all outcomes) than any of the other 3 combinations; conversely, no significant differences in terms of outcomes were observed among the remaining 3 groups, which could, therefore, be collapsed into a single subgroup (hereafter referred to as hybMENA negative) (Fig. S5). At multivariate analysis, hybMENA remained a highly significant independent predictor of outcome (data not shown); Kaplan-Meier curves according to hybMENA are shown in Fig. 3G-I; 3- and 5-year outcomes are summarized in Table S4.

### Risk class generation

**Figure 4:**
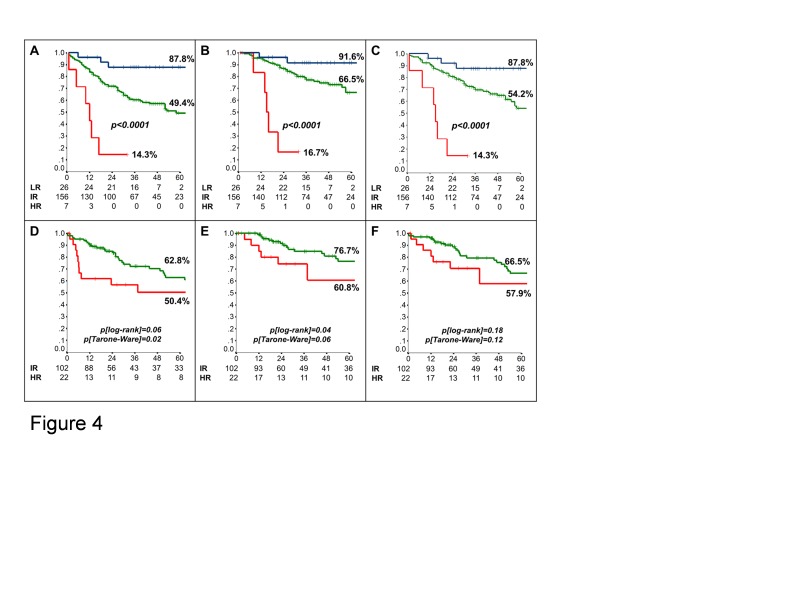
Risk class-based prognostic model Kaplan-Meier analysis of (A) Disease-Free- (DFS) (B) Cancer-Specific (CSS) and (C) Overall-Survival (OS) of patients included in the training set according to the generated risk classes (LR: Low risk [blue line]; IR: Intermediate risk [green line]; HR: High risk [red line]). (D-F) Kaplan-Meier analysis of DFS (D), CSS (E) and OS (F) of patients included in the external validation set.

Next, we generated risk classes according to the following combinations of clinical/molecular factors identified at multivariate analysis: 1) Low-Risk (T1, number of resected lymph nodes≥10, and hybMENA positive); 2) High-Risk (T>1, number of resected lymph nodes<10, and hybMENA negative); 3) Intermediate-Risk (any other combination). The derived 3-risk class survival model strikingly discriminated between patients at different risk of relapse, cancer-related death, and death for any cause (Fig. 4A-C and Table S4). The accuracy of the model was 61% (standard error 0.03, p=0.01), according to ROC analysis.

Finally, we externally validated the 3-risk class survival model in an independent dataset of 133 early-stage, N0, NSCLC patients who underwent curative surgery at the University of Perugia; relevant patient characteristics are shown in Table S5. Stratification according to risk classes significantly discriminated between patients at Intermediate- and High-Risk of relapse and cancer-related death in the validation set as well (Fig. 4D-F); despite an appreciable separation of the curves, OS differences did not reach statistical significance, due to the low number of events (36 deaths). Only 4 patients in the validation dataset were grouped as Low-Risk and therefore the Low-Risk group could not be considered in the external validation dataset.

## DISCUSSION

We analyzed hMENA isoform expression in NSCLC by biochemical, functional and immunohistochemical methodologies and we found that alternative expression of hMENA isoforms represents a prognostic factor in radically resected NSCLC and usefully complements clinical parameters to accurately predict individual patient risk of relapse and death.

According to our experimental data, the hMENA^11a^ isoform is expressed only in a subset of NSCLC cell lines showing an epithelial phenotype. Conversely, the lack of this isoform and the expression of hMENAΔv6 is associated with an invasive, ‘mesenchymal’ phenotype. This effect is related to the ability of hMENA^11a^to remodel the actin cytoskeleton towards an epithelial-like cytoarchitecture, similar to observations in breast cancer cells [14]. The differential expression of hMENA isoforms in NSCLC may also impact on other aspects of the malignant phenotype. Alterations in cell cytoskeletal organization can impact on cell shape and functional competence through proliferative as well as invasive characteristics; indeed, hMENA^11a^ impacts cell morphology as shown in 2D and 3D cultures and is implicated in a non-invasive, but proliferative behavior in agreement with previous results in breast cancer [16].

From a clinical perspective, the distinct functions of hMENA isoforms and their lack of expression in normal lung tissue support a possible involvement of hMENA overexpression and splicing in NSCLC carcinogenesis and progression and suggest that differential isoform expression could constitute a potential marker of aggressiveness in early stage NSCLC. Based on preclinical results obtained in cell line models, we can speculate that the group of tumors that are pan-hMENA high/hMENA^11a^ low would include the tumors that express the hMENAΔv6 isoform, although this cannot be directly assessed, due to the lack of IHC-validated antibody.

Recently, combined genomic and transcriptomic analysis has shown that cancer-associated splicing events frequently occur in lung cancer, suggesting that differential isoform expression between normal and cancer samples may represent a potentially novel biomarker [19]. Furthermore, the alternative expression of splicing isoforms could be a reliable marker of tumor progression and response to therapy [17, 20].

Here we present a prognostic algorithm based on differential hMENA isoform expression, which uses a simple combination of IHC staining for two individual parameters (pan-hMENA and hMENA^11a^) and readily available clinical factors (T-size and the number of resected lymph nodes) to accurately segregate groups of patients with a 5-yr risk of relapse or death ranging from 12% to 76%. A possible prognostic value of the relative expression of different hMENA isoforms has been recently suggested in breast cancer: normalized AQUA scores for pan-hMENA and hMENA^11a^ were used to calculate a hybrid variable (Menacalc fraction) by subtracting the z score of hMENA^11a^from the z score of pan-hMENA [21]. Methodologically, the approach we took to establish the prognostic value of pan-hMENA and hMENA^11a^ expression followed a straightforward protocol to establish prognostic algorithms [22]. Interestingly, one of the independent prognostic parameters (number of resected lymph nodes) had been previously identified by our group, using a similar methodological approach [23]. The prognostic performance of the derived risk-class model has been externally validated in an independent clinical series, although the low-risk class could not be analyzed due to the low number of patients (Fig. 4D-F). Even though the overall prognosis of the validation cohort was slightly more favorable (only T1 and T2 cases), the risk-class model maintained some discriminating power between the Intermediate and High Risk classes. Such prognostic performance is similar to that reported for the Malignancy-Risk signature, arguably the most robust prognostic gene signature reported so far, in stage I NSCLC [24], although such a signature only applies to adenocarcinoma or non-squamous NSCLC.

Our model has particular relevance for early stage NSCLC: indeed, CT-scan based screening techniques have led to a significant increase in the proportion of NSCLC cases diagnosed in stage I-II, but platinum-based adjuvant chemotherapy has not significantly improved these patients' survival. Recent data suggest that patients classified at high risk of relapse by gene signatures may actually benefit from adjuvant treatment regardless of stage [24], although the retrospective nature of these studies precludes the possibility to analyze the relative impact of pathological staging in this context.

With respect to currently available models, our study has two main strengths: 1) while few other experiences have specifically looked at prognostic determinants in early stage, node-negative disease, we elected to look at the prognostic impact of the differential expression of hMENA isoforms selectively in N0 patients; 2) while most other prognostic studies have attempted to demonstrate a dominant role of biology (in terms of expression of prognostic signatures) over clinical staging [24, 25], we have integrated clinico-pathological staging (tumor size), standard surgical approaches (number of resected lymph nodes), and biological variables (differential hMENA isoform expression) into a coherent algorithm for overall risk assessment. This approach is justified by the fact that each prognostic feature has been shown to independently contribute to the overall prognosis, as demonstrated by multivariate analysis, and potentially will enable clinicians to incorporate all relevant prognostic features into a relatively simple and practical prognostic algorithm. The major limitation of the study presented herein is its retrospective nature and the fact that, as adjuvant chemotherapy is not routinely recommended in stage I disease (accounting for 203/248 patients included in the training set), only 27 patients in our dataset had received adjuvant chemotherapy, thus precluding the evaluation of a possible predictive value of our proposed risk-score system. Therefore, whether adjuvant chemotherapy is able to significantly counteract the risk of recurrence and death in node-negative NSCLC patients that are classified at intermediate/high risk by the proposed prognostic algorithm will need to be prospectively assessed in separate studies.

In conclusion, our data strongly support the inclusion of hMENA splicing-related biomarkers in the prognostic assessment of early-stage NSCLC. This may pave the way to more effective patient selection for adjuvant studies and possibly yield novel, potentially druggable, therapeutic targets.

## METHODS

### *In vitro* studies

Cell lines were purchased from American Type Culture Collection (ATCC, Rockville, MD) and cultured in RPMI 1640 medium (Gibco, Invitrogen, Pisley, UK) supplemented with 10% inactivated fetal bovine serum at 37°C in 5% CO_2_-95% air. All cell lines were routinely morphologically checked by microscope, growth curve analysis by 3H-Thymidine incorporation assay and Mycoplasma detection (Roche, Monza, Italy). Western blot analysis, cell invasion assays, and immunofluorescence were performed using established techniques (see Supplementary Materials for details). For transfection studies, exponentially growing cells were plated in 6-well plates at a density of 3×10^5^ cells/well; after 24 h cells were transfected with 1,5 μg/ml *hMENA ^11a^*, *hMENAΔv6* cDNA, or with vector alone (pcDNA3) using LipofectAMINE2000 (Invitrogen, Carlsbad, CA). Calu1 cells used for 3D cultures were transfected in suspension before plating. Briefly, 4×10^5^ detached cells were incubated, in 15 ml tubes in 2ml culture medium containing 3μg of cDNA and 5μl of LipofectAMINE 2000 and shaken by hand every 30min. After 5h cells were washed and half of them were plated and cultured for 72h in six-well plates and then evaluated for transfection efficiency by Western blot. The other half were seeded on top of a thin layer of polymerized growth factor reduced reconstituted basement membrane (Matrigel; BD Pharmingen) in the presence of growth medium containing 5% (vol/vol) Matrigel. After 72h cells were analysed by phase-contrast microscopy.

### Patient population

All NSCLC patients resected with curative intent at the Regina Elena National Cancer Institute between 2001 and 2006 and without pathological lymph-node involvement (N0) were considered eligible for the prognostic analysis (training set, Table S2). Follow-up data were obtained from hospital charts and by corresponding with the referring physicians, analyzed, and reported according to Shuster et al. [26]. External validation was accomplished using a series of 133 consecutive, node-negative, NSCLC patients who underwent surgery with curative intent at the University of Perugia (validation set, Table S5). The study was reviewed and approved by the ethics committee of the Regina Elena National Cancer Institute, and written informed consent was obtained from all patients.

### Tissue microarray construction and molecular analyses

For immunohistochemical (IHC) analyses, Tissue Micro Arrays (TMA) were constructed from the original formalin fixed, paraffin embedded (FFPE) blocks. Two representative tumor areas were carefully selected on routine haematoxylin/eosin-stained sections. Two core cylinders (1 mm diameter) were taken and deposited in separate recipient paraffin blocks using a specific arraying device (Alphelys, Euroclone, Milan, Italy). In addition to NSCLC tissue, the recipient block also received normal lung tissue and cell line pellets as negative and positive controls, respectively. In cases where informative results on TMA were absent due to missing tissue, no tumor tissue, or unsuccessful staining or hybridization, we re-analyzed the correspondent routine tissue section. Three-micron sections of the resulting microarray blocks were made for immunohistochemistry (IHC) assays, carried out as described in the Supplementary Methods. Immunostained slides were analyzed and scored independently by 2 different investigators (M.Mo. and P.V.), blinded to the clinical data. Genetic analysis of the *EGFR* gene was carried out as previously described [27].

Staining for pan-hMENA and hMENA^11a^ was quantified in terms of both staining intensity score and percent of positive cells for each individual case (as detailed in the Supplementary Methods); continuous variables were then generated as the product of the staining intensity score and the percentage of positive cells [27], thereby obtaining a single numerical value ranging from 0 to 300. The value was calculated individually for each TMA copy; the mean of the two separate copies were compared by parametric and non-parametric tests for paired samples, in order to find potential differences and to obtain a single variable for cut-off analysis [28]. To check the functional form of pan-hMENA and hMENA^11a^ continuous variables, Martingale residual plot (MRP) analysis was used; in the presence of non linear distribution of ratios, optimal cut-off points were identified by maximally-selected log rank statistics (Fig. S4A-B) and confirmed by classification and regression tree (C&RT) and ROC analysis [23]. Finally, we created a hybrid, dichotomized variable, taking into account the relative expression of pan-hMENA and hMENA^11a^ (as detailed in the Results and in Fig. S5); such dichotomized variable (hybMENA, positive vs negative) was then employed for all other analyses.

### Statistical analysis

To assess the prognostic relevance of hMENA isoforms, a stepwise protocol to build a nomogram for cancer prognosis was followed, according to Iasonos et al. [22]; the same methodology had been used previously by our group to establish the prognostic role of the number of resected lymph nodes in early NSCLC [23]. Hazard ratios (HR) and 95% confidence intervals (95% CI) were estimated for each variable using the Cox univariate model [29]; a multivariate Cox proportional hazard model was developed using stepwise regression (forward selection, enter/remove limits p=0.10 and p=0.15 respectively), in order to identify independent predictors of outcomes; potential interactions between significant variables were taken into account when developing the multivariate model. Internal model validation was obtained by bootstrap resampling analysis [22, 30]. Based on the developed multivariate models, a logistic equation including the coefficients of the regression analysis was constructed to estimate individual patient probability (IPP) of outcome (at pre-specified time points): probability of event = (Exp∑(X x Beta) + intercept(alfa))/(1+(Exp∑(X x Beta) + intercept(alfa))), where X x Beta is the coefficient Beta for each single confounding factor X [31]. Receiver operating characteristic (ROC) curve analysis was carried out to assess the predictive accuracy of prognostic models [32]. Disease-free, cancer-specific, and overall survival (DFS/CSS/OS) were calculated by the Kaplan-Meier product limit method from the date of the surgery until relapse or death [33]. The log-rank and Tarone-Ware tests were used to assess differences between subgroups. Significance was defined at the p<0.05 level. The SPSS® (21.0), R® (2.6.1), SAS® (9.0) and MedCalc® (12.7.5) statistical programs were used for all analyses.

## SUPPLEMENTARY MATERIAL FIGURE AND TABLE


