# Supplementary material for: Prognostic impact of alternative splicing-derived hMENA isoforms in resected, node-negative, non-small-cell lung cancer
Source: Oncotarget. 2014 Oct 21;5(22):11054–63. doi: 10.18632/oncotarget.2609 (PMC4294370; doi:10.18632/oncotarget.2609)
Supplement: Supplementary file 1 [file oncotarget-05-11054-s001.pdf]

Prognostic impact of alternative splicing-derived hMENA isoforms in resected, node-negative, non-small-cell lung cancer

Supplementary Material

Western blot analysis. Cells were lysed as reported [14]. Lysates (30µg or 50µg) were resolved on 10% polyacrylamide gel and transferred to nitrocellulose membrane as described. Blots were probed with the following antibodies: 10µg/ml of pan-hMENA rabbit CKLK1 antibody; anti-hMENA^{11a} (1µg/ml) and anti-hMENAΔv6 (0,6µg/ml) [14]; mouse anti-E-CADHERIN from BD Biosciences (San Jose, CA); in 3% skimmed milk/TBST overnight at 4°C. For actin signal, blots were reprobed with monoclonal anti-ACTIN, mouse-ascites Fluid clone AC-40 (Sigma Aldrich, Poole, UK).

Cell invasion assay. Forty-eight hours after transfection, cells were counted and equal numbers (25.000 A549 and 50.000 H1975 cells) were seeded in Matrigel invasion chambers (24 wells; BD Biocoat Matrigel invasion chamber, BD Biosciences) in duplicate following the manufacturer's instructions. Cells were allowed to invade for 24h, then stained and photographed and at least 10 fields were counted. Each experiment was performed three times.

[^{3}H] Thymidine Incorporation Assay. The day after transfection cells from each well were transferred to 4 wells of 48-well-plates. After 48 hrs ^{3}H-thymidine (Perkin Elmer Life and Analytical Sciences, Boston, MA) was added at 5 mCi/mL for 4 h. Following medium removal, cells were washed twice with cold PBS, treated with 10% trichloroacetic acid for 30 min at 37°C, solubilized with 0.4 N NaOH and counted for incorporation of ^{3}H on β liquid scintillation counter in 5 mL of scintillation fluid. Each experiment was done in quadruplicate and results were expressed as the means of at least three separate experiments.

Immunofluorescence. Cells were cultured on collagen I precoated coverslips in individual wells of a 24-well plate for 48h. Cells were fixed in modified paraformaldehyde fixative (60mM PIPES, 25mM HEPES, 10mM EGTA, 2mM MgCl2, 120mM sucrose, 4% paraformaldehyde; final pH 7.4) for 15 minutes at room temperature. Cells were permeabilized with 0,2% Triton X100 for 10 min, incubated with 1% BSA/PBS for 1h and then incubated with pan-MENA mAb (kindly provided by Prof. Frank Gertler, Massachusetts Institute of Technology, Cambridge, MA) according to standard protocols, and were stained using AlexaFluor568-phalloidin, AlexaFluor488-conjugated donkey anti-mouse antibody, and DAPI (Invitrogen, Carlsbad, CA) then were streaked on slides and mounted in Vectashield hard set media (Vector labs, Burlingame, CA). Fixed and stained cells were imaged using a DMIRE (Leica Microsystem) microscope with FW4000 software.

Surgical procedures. Complete lympho-adenectomy was routinely performed in all the radical resections for NSCLC, as previously described [23]. Complete mediastinal lymphatic dissection has been performed as described by Cahan and revised by Martini and Naruke. By adopting this approach, the mediastinal space is completely exposed up to the highest stations and station 7 is explored up the controlateral bronchus.

Immunohistochemistry. The pan-hMENA monoclonal antibody (clone 21; BD Transduction), that specifically recognizes all of the hMENA isoforms, was used as previously reported [14]; hMENA^{11a} expression was assessed using the anti-hMENA^{11a}–specific monoclonal antibody produced and validated by our group [14]. The following antibodies were also used for immunostaining: anti-HER2 A0485 (polyclonal, Dako Milan, Italy), anti-ERα 6F11 (monoclonal, Novocastra, Leica, Milan Italy), anti-ERβ1 14C8 (monoclonal, Genetex, Prodotti Gianni, Rome, Italy), anti-ERβ2 57/3 (monoclonal, Serotec, Space, Milan, Italy), anti-E-cadherin 36 (BD Transduction, San Jose, CA), anti-Vimentin V9 (monoclonal, Dako), anti-p-AKT/Ser473 736E11 (monoclonal, Cell Signaling, Euroclone, Milan, Italy). Immunoreactions for HER2, ERβ2,

Vimentin, E-cadherin, and $hMENA^{11a}$ were revealed by a streptavidin-biotin enhanced immunoperoxidase technique (Super Sensitive MultiLink, Leica) in an automated autostainer (Bond III, Leica). Immunoreactions for p-AKT and ERβ1 were revealed after an overnight incubation of the primary antibody. EGFR expression was detected using the EGFR PharmDX kit (Dako).

For hMENA and $hMENA^{11a}$ both percentage of stained tumor cells and intensity score (from 0 to 3+) were taken into account. HER2 and EGFR IHC positivity was scored as follows: score 0 as negative and scores 1+, 2+ and 3+ as positive. ERα, ERβ1, ERβ2 were considered IHC positive when >10% of the neoplastic cells showed distinct nuclear immunoreactivity. Vimentin and p-AKT were scored as positive when tumor cells, displayed a strong immunoreactivity independent of the percentage of stained cells. The E-cadherin expression level was determined in comparison with normal lung structures present in the peritumoral tissue and scored as normal, reduced, or absent. Normal staining was defined when ≥70% of the tumor cells presented a strong membranous staining, whereas reduced expression was defined when <70% of tumor cells presented a weak membranous staining. No stained cells were observed in the negative cases.

Statistical methods. Harrell's guidelines were followed to establish the maximum number of covariates that could be reliably assessed based on the number of observed events (86 death events at the time of the analysis). Correlations between clinic-pathological and bio-molecular variables were analyzed according to Chi-square, T-student and Mann-Whitney (non parametric) tests. In order to address the multivariate model overfit and to validate the results [22, 23], a cross validation technique, which evaluates the replication stability of the final Cox multivariate model in predicting all outcomes, was employed using a re-sampling procedure considering variables that proved independent at multivariate analysis for at least one outcome. This technique generates a number of simulation datasets (at least 100, each approximately 80% of the original dataset size), by randomly selecting patients from the original sample, in order to establish the consistency of the model across

less powered patient samples [22]. The cross validation allows to test the accuracy of the multivariate model developed, as assessed by ROC analysis [30].

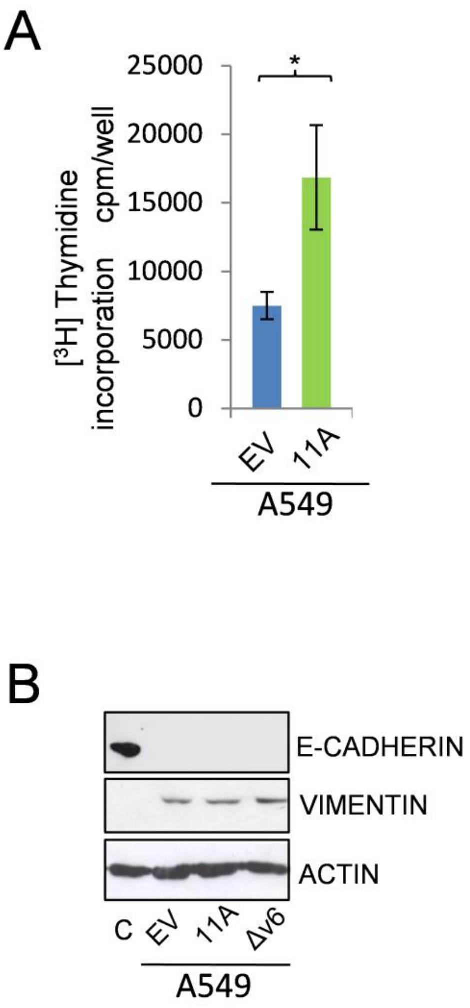

Supplementary Figure 1 : A) Proliferation assay of A549 cells transfected with the empty vector (EV) or hMENA^{11a} (11A) was conducted 72 h after the cDNA transfection by measuring [^{3}H] thymidine incorporation as described in Supplementary Materials and Methods. Columns, the mean of three different experiments; bars, SD. * Significantly different as determined by Student t tests $p < 0.05$. **B**) WB analysis of A549 cells transfected with the empty vector (EV), hMENA^{11a} (11A) or hMENAΔv6 (Δv6), with E-CADHERIN and VIMENTIN Abs. MCF7 cells were used as positive control of E-Cadherin expression (C).

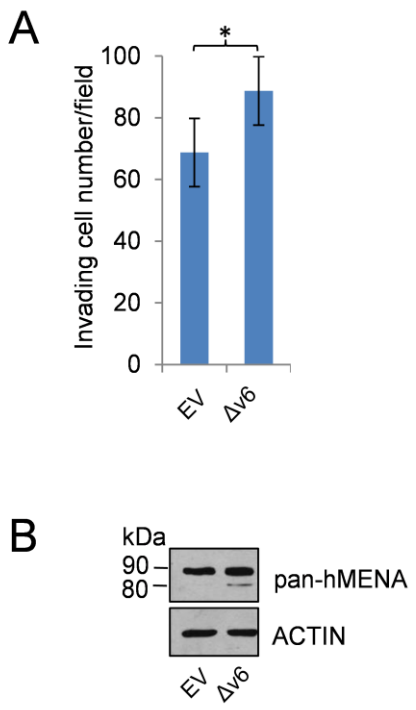

Supplementary Figure 2: A) Matrigel invasion assays of H1975 cells transfected with the empty vector (EV) or hMENAΔv6 (Δv6). The ability of invasion has been measured by the use of Matrigel coated transwell filters toward a gradient of serum. The assay was repeated three times and performed in triplicate each time. *p= 0.059. **B)** WB analysis using pan-hMENA Ab of H1975 cells transfected with the empty vector or hMENAΔv6, shows the 80kDa band corresponding to hMENAΔv6.

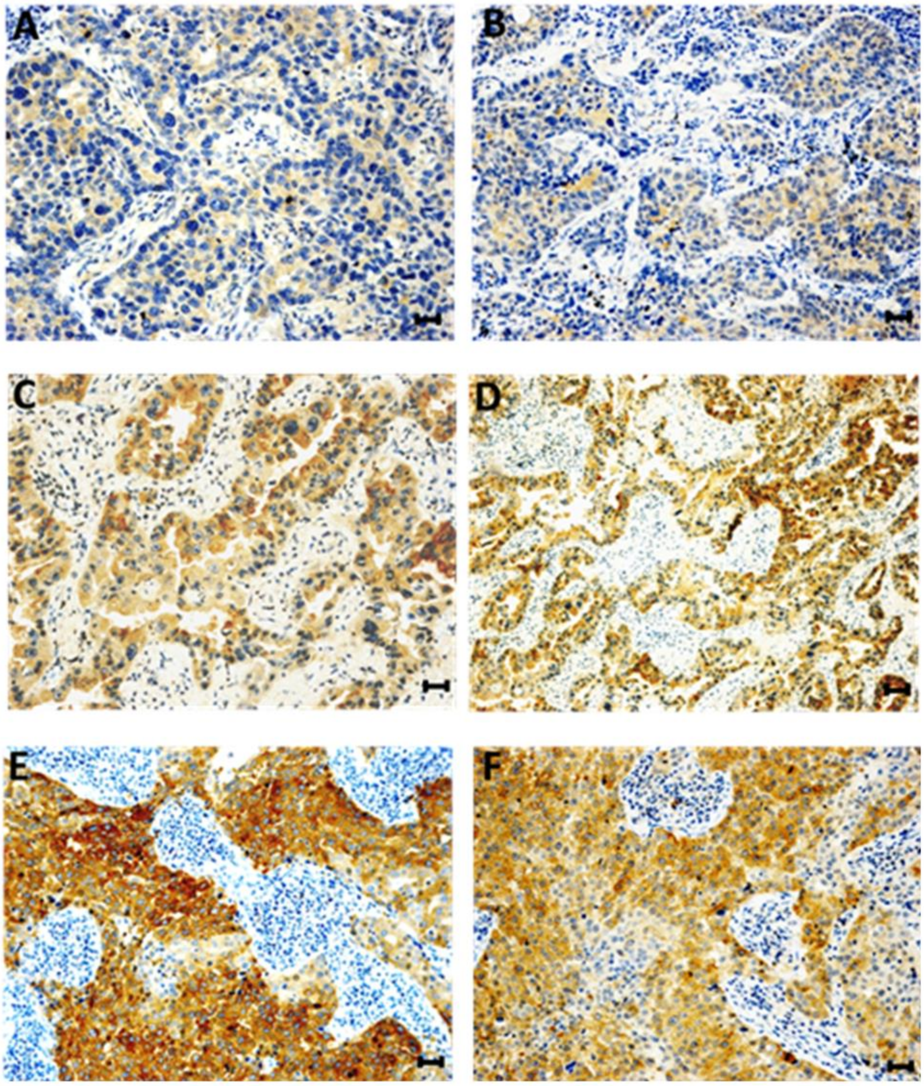

Supplementary Figure 3 : Representative immunohistochemical staining of three lung carcinomas showing overlapping pan-hMENA and hMENA^{11a} expression score 1 (**A-B**); 2 (**C-D**); 3 (**E-F**) respectively. Magnification 20X. Scale bar 30 µm.

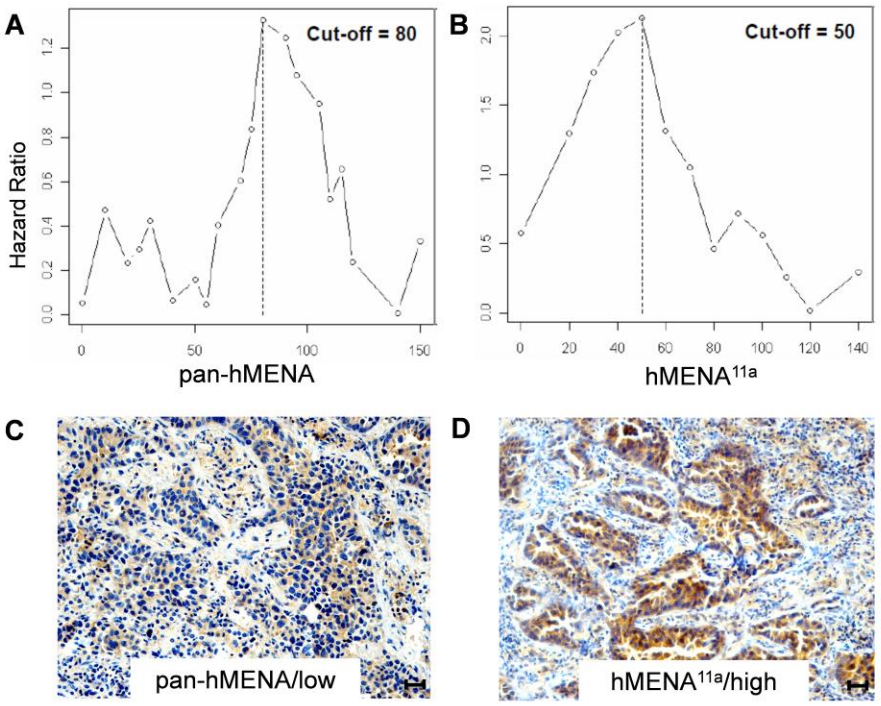

Supplementary Figure 4 : Cut-off selection for dichotomized pan-hMENA (**A**) and hMENA^{11a} (**B**) immunostaining; optimal cut-offs were identified by maximally selected log-rank statistics. A representative NSCLC case with low pan-hMENA **(C)** and high hMENA^{11a} (**D**) staining as detected by IHC. Magnification 20X. Scale bar 30 µm.

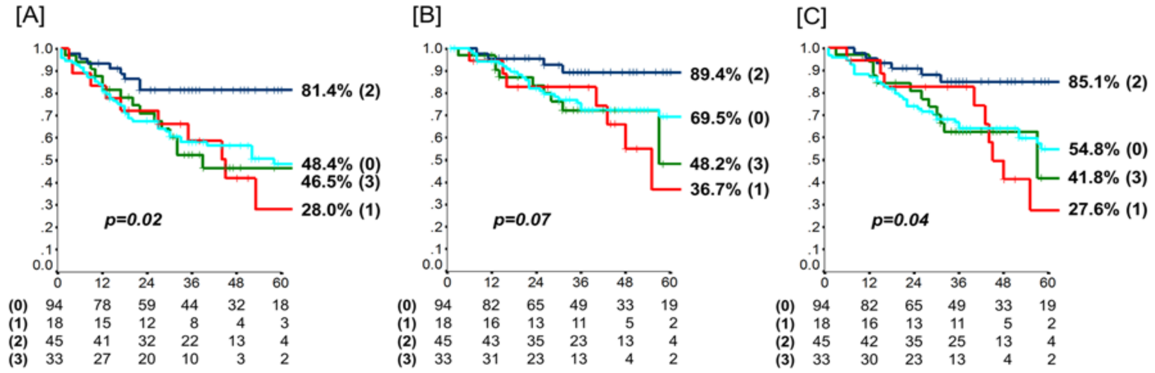

Supplementary Figure 5 : A) Kaplan-Meier analysis of Disease-Free- (DFS) , **B**) Cancer-Specific- (CSS), and **C**) Overall-Survival (OS) of resected, node-negative, NSCLC patients included in the training set, according to the four possible combinations of high and low expression of pan-hMENA and $hMENA^{11a}$. Patients with low pan-hMENA/high-$hMENA^{11a}$ were classified as hybrid MENA (hybMENA) positive (group 2: blue line); patients with all other combinations (groups 0, 1, 3) were classified as hybMENA negative.

Supplementary Table 1: Characteristics of lung cancer cell lines used

Cell Line	Tumor Type	Mutational status			Gender	Site
		TP53	*KRAS*	*EGFR*		
A427	AC	Wt	Mt	Wt	M	
A549	AC	Wt	Mt	Wt	M	
AE2	SCLC	-	-	-	M	metastatic site
Calu1	AC	Mt	Mt	Wt	M	metastatic site; pleura
H1650	AC	Wt	Wt	Mt	M	metastatic site
H1975	AC	Wt	Wt	Mt	F	
H460	AC	Wt	Mt	Wt	M	metastatic site

Legend Supplementary Table 1. AC, Adenocarcinoma; SCLC, Small Cell Lung Cancer; Mt, mutant; Wt, wild type

Supplementary Table 2: Patients' Characteristics (training set).

Factor	N°	%
**Age**		
Median (years)	67	*n.a.*
Range	28-83	
**Sex**		
Male	177	*71.4*
Female	71	*28.6*
**Surgery**		
Lobectomy	224	*65.3*
Bilobectomy	10	*23.8*
Pneumonectomy	14	*10.9*
**T-size***		
T1a	93	*37.5*
T1b	110	*44.4*
T2	23	*9.7*
T3	10	*4.0*
T4	12	*4.8*
**Grading**		
G1	13	*5.2*
G2	97	*39.1*
G3	127	*51.2*
Unknown	11	*4.4*
**Histology**		
Adenocarcinoma	126	*50.8*
Squamous	92	*37.1*
Other	30	*12.1*
**RN**		
≤10	84	*34*
>10	164	*66*
**EGFR Mutation**		
Wild Type	199	*80.2*
Mutant	19	*7.7*
Not determined	30	*12.1*
**Stage***		
IA	93	*37.5*
IB	110	*44.0*
II	23	*9.7*
III	10	*4.0*
IV	12	*4.8*

Legend Supplementary Table 2. N°: number of patients; %: rate of patients; n.a.: not applicable; T-size: tumor size; RN: number of resected nodes. *According to the TNM classification of malignant tumors, 7th edition.

Supplementary Table 3: No significant associations between Low pan-hMENA and High hMENA^{11a} with clinico-pathological variables.

	N° pts	Low pan-hMENA		High hMENA^{11a}	
		**n. (%)**	***p-value***	**n. (%)**	***p-value***
**Sex**					
Male	144	104 (72.2)	*0.43*	61 (42.7)	*0.76*
Female	58	45 (77.6)		23 (40.4)	
**Surgery**					
Lobectomy	184	137 (74.5)		78 (42.9)	
Bilobectomy	8	5 (62.5)	*0.72*	3 (42.9)	*0.60*
Pneumonectomy	10	7 (70.0)		3 (27.3)	
**Histology**					
Adenocarcinoma	100	78 (78.0)		48 (47.1)	
Squamous	74	54 (73.0)	*0.18*	27 (37.8)	*0.31*
Other	28	17 (60.7)		8 (33.3)	
**Grading**					
1-2	91	73 (80.2)	*0.08*	42 (48.3)	*0.09*
3	108	75 (69.4)		40 (36.4)	
**T-size***					
T1a-T1b	169	124 (73.4)	*0.57*	74 (44.6)	*0.08*
T2-T4	32	25 (78.1)		9 (28.1)	
**RN**					
≤10	67	48 (71.6)	*0.63*	32 (46.4)	*0.36*
>10	135	101 (74.8)		52 (39.7)	

Legend Supplementary Table 3. N°: number; pts: patients; n.: number of patients; T-size: tumor size; RN: number of resected nodes. *According to the TNM classification of malignant tumors, 7th edition.

Supplementary Table 4: Three and five-year outcomes.

Outcome	Variable	3-yr Outcome	5-yr Outcome	*p-value*
**DFS**	pan-hMENA			
	Low	63.2%	53.5%	*0.17*
	High	54.6%	34.6%	
	hMENA[11a]			
	High	69.5%	67.4%	*0.03*
	Low	58.9%	45.2%	
	**hybMENA**			
	hybMENA+	81.5%	81.5%	*0.003*
	hybMENA-	57.2%	44.7%	
	**Risk Classes**			
	Low	87.8%	87.8%	
	Intermediate	60.3%	49.4%	*<0.0001*
	High	14.3%	-	
**CSS**	pan-hMENA			
	Low	77.7%	72.2%	*0.08*
	High	74.7%	39.7%	
	hMENA[11a]			
	High	83.2%	76.8%	*0.12*
	Low	65.1%	63.6%	
	**hybMENA**			
	hybMENA+	89.4%	89.4%	*0.02*
	hybMENA-	73.6%	62.4%	
	**Risk Classes**			
	Low	91.6%	91.6%	
	Intermediate	77.2%	66.5%	*<0.0001*
	High	30.0%	-	
**OS**	pan-hMENA			
	Low	69.6%	59.5%	*0.16*
	High	68.6%	31.6%	
	hMENA[11a]			
	High	75.4%	69.6%	*0.056*
	Low	68.0%	50.2%	
	**hybMENA**			
	hybMENA+	85.1%	85.1%	*0.006*
	hybMENA-	65.9%	48.9%	
	**Risk Classes**			
	Low	87.6%	87.6%	
	Intermediate	69.7%	54.2%	*<0.0001*
	High	14.3%	-	

Legend Supplementary Table 4. DFS: disease free survival; CSS: cancer specific survival; OS: overall survival.

Supplementary Table 5: Patients' characteristics (validation set).

Factor	N°	%
**Age**		
Median (years)	67	*n.a.*
Range	40-84	
**Sex**		
Male	112	*84.2*
Female	21	*15.8*
**T-size***		
T1	73	*54.8*
T2	60	*45.1*
**Histology**		
Adenocarcinoma	55	*41.4*
Squamous	57	*42.9*
Other	21	*15.8*
**RN**		
≤10	51	*38.3*
>10	82	*61.7*

Legend Supplementary Table 5. N°: number of patients; %: rate of patients; n.a.: not applicable; T-size: tumor size; RN: number of resected nodes. *According to the TNM classification of malignant tumors, 7th edition.
